# Essential Role of Integrin-Linked Kinase in Keratinocyte Responses to Mechanical Strain

**DOI:** 10.3390/ijms27062858

**Published:** 2026-03-21

**Authors:** Alena Rudkouskaya, Iordanka A. Ivanova, Samar Sayedyahossein, Lina Dagnino

**Affiliations:** 1 Department of Physiology and Pharmacology, Western University, London, ON N6A 5C1, Canada; rudkoua@amc.edu (A.R.); danyivanova@hotmail.com (I.A.I.); samaryahossein@vtc.vt.edu (S.S.); 2 Children’s Health Research Institute, London, ON N6C 2V5, Canada; 3 Department of Oncology, Western University, London, ON N6A 5W9, Canada

**Keywords:** epidermis, keratinocytes, ILK, mechanotransduction

## Abstract

Mechanical signals play key roles in the regulation of epidermal homeostasis and regeneration after injury. Integrins are key components of focal adhesions, and these complexes are major contributors to mechanotransduction. In keratinocytes, integrin-linked kinase (ILK) modulates essential processes for epidermal homeostasis and wound repair. However, its functions in the transduction of mechanical stimuli have remained virtually unexplored. In this study, we characterized epidermal tissues and primary keratinocytes from mice with epidermis-restricted inactivation of the *Ilk* gene (ILK-KO). ILK-deficient epidermis exhibits abnormalities in key components of mechanotransduction cascades, including disruptions in hemidesmosomal Collagen XVII immunoreactivity at the dermal–epidermal junction, and marked reduction in the nuclear localization of the mechanosensitive transcriptional regulator YAP. In wild-type (ILK+), but not in ILK-KO-cultured keratinocytes, exposure to cyclic bidirectional strain induced marked F-actin cytoskeletal rearrangements, characterized by the assembly of thick cortical actin bundles and stress fibers, as well as YAP nuclear translocation and transcriptional activity. Exposure to mechanical strain was additionally accompanied by differential changes in miRNA expression between ILK+ and ILK-KO cells. These findings reveal multiple and previously unappreciated key regulatory roles for ILK in epidermal keratinocyte responses to mechanical signals.

## 1. Introduction

The skin fulfills unique functions, serving as a critical barrier that separates the organism from its external environment. The epidermis of the skin must respond to a wide variety of chemical, biological and physical agents, including internal and external mechanical stimuli. In vivo, stretch and/or tensile strength are the main forms of mechanical signals that occur in epidermal keratinocytes. Moderate physiological cutaneous stretch occurs during pre- and postnatal growth, and this property has been harnessed to improve wound healing and skin reconstruction [1]. In contrast, dysregulated mechanical stress can lead to inflammatory disorders, fibrosis and other skin pathologies [2].

In keratinocytes and other cell types, integrins are central for mechanosensing and for transduction of mechanical forces into biochemical responses [3]. Integrins bind to extracellular matrix (ECM) substrates, associate with linker proteins, such as integrin-linked kinase (ILK), kindlins and focal adhesion kinase, and activate signaling pathways to transmit mechanical forces to the actin cytoskeleton [3].

ILK is a pseudokinase scaffold protein that plays important roles in focal adhesion assembly. In this manner, ILK regulates F-actin organization, cell polarity and migration [4]. ILK fulfills key functions necessary for multiple aspects of epidermal function. For example, ILK is essential for hair follicle morphogenesis and epidermal barrier formation, as well as keratinocyte stem cell activation and ECM deposition during wound healing [5,6,7,8,9]. Transcriptomic profiling of ILK-deficient epidermis has revealed unexpected potential roles in melanocyte development, pigmentation, and the regulation of homeobox genes [10]. Thus, ILK is not only a structural adaptor, but also a regulator of extracellular matrix composition and stem cell niche signaling. How all these functions and pathways integrate with keratinocyte mechanotransduction remains unclear.

Downstream targets of integrin engagement are the mechanosensitive transcriptional coactivators YAP and TAZ [11]. YAP/TAZ activation is regulated in response to changes in cytoskeletal F-actin tension, which in turn depends on mechanical stimuli [12]. In response to increased substrate stiffness or strain, YAP/TAZ translocate from the cytoplasm to the nucleus, activating transcriptional programs that promote proliferation and maintenance of stemness in keratinocytes and other cell types. YAP/TAZ exert their effects primarily through interaction with TEAD transcription factors, modulating epidermal lineage commitment, and integrating mechanical signals with the Notch and Wnt pathways to maintain epidermal homeostasis [12,13]. Increased ECM stiffness or sustained mechanical tension additionally activate YAP/TAZ-mediated transcription of mechanosensitive miRNAs, thus allowing fine-tuning of cellular responses to mechanical signals at the post-transcriptional level [14].

Despite the recognized role of ILK as a central mediator of integrin-activated pathways, its role as a mechanosensory modulatory hub remains largely unexplored. Here we have investigated whether ILK links mechanical signals to cytoskeletal organization and transcriptional programs essential for keratinocyte function and epidermal homeostasis. Our findings indicate that ILK modulates processes activated in response to mechanical strain at multiple levels, including F-actin regulation, transcriptional programs involving YAP activation and changes in microRNA expression.

## 2. Results

### 2.1. Alterations in Epidermal Integrity and Organization in ILK-KO Epidermis

**Figure 1:**
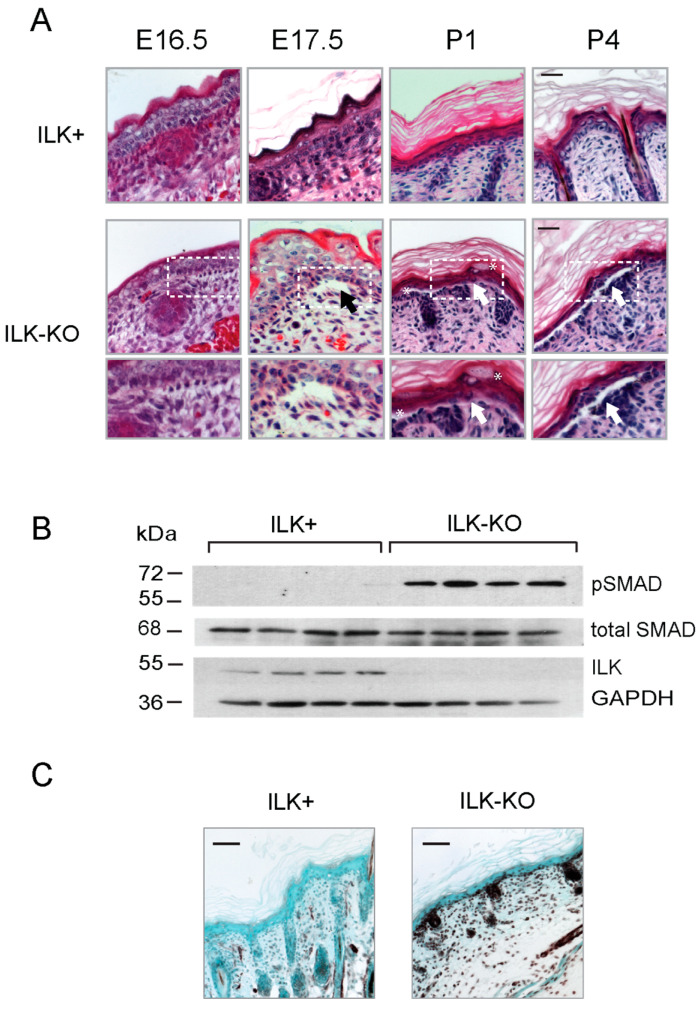
Altered integrity in ILK-KO epidermis. (**A**) Representative micrographs showing epidermal blisters in ILK-KO epidermis. Tissue sections of mice with the indicated phenotypes and ages were stained with hematoxylin and eosin. Boxed areas are shown at higher magnification below each micrograph and the arrows indicate blistered areas. Asterisks show regions with impaired epidermal integrity. Bar, 50 μm. (**B**) Epidermal lysates from individual ILK+ and ILK-KO littermates were analyzed by immunoblot with antibodies against phosphorylated (pSMAD), total SMAD 2/3 or ILK. GAPDH was used as loading control. (**C**) Representative micrographs of skin sections from 3-day-old ILK+ or ILK-KO mice immunostained with antibodies against α-smooth muscle actin and counterstained with methylene green. Bar, 50 μm.

ILK is a key scaffold protein that contributes to focal adhesion assembly, thus participating in integrin-mediated cell attachment to the extracellular matrix (ECM) [15]. We first investigated the consequences of targeted inactivation of the *Ilk* gene on keratinocyte adhesion to the underlying basement membrane, at the dermoepidermal junction. Histological analysis of cutaneous tissues revealed the presence of dermal–epidermal blistering in perinatal mice with epidermis-specific *Ilk* gene inactivation (hereafter termed ILK-KO), particularly in areas subjected to friction. Significantly, microblisters were also detected in some epidermal areas in embryos as early as 16.5 days of gestation, suggesting an intrinsic impairment of epidermal adhesion and skin fragility in the absence of ILK (Figure 1A). These abnormalities in ILK-KO epidermis were also accompanied by activation of pathways involving Transforming Growth Factor-β (TGF-β) signaling, as evidenced by increases in the abundance of phosphorylated SMAD 2/3 proteins, and upregulation of α-smooth muscle actin in the skin of these animals (Figure 1B,C). This suggests the possibility of activation of cutaneous wound repair pathways upon tissue disruption resulting from epidermis-restricted *Ilk* gene inactivation.

**Figure 2:**
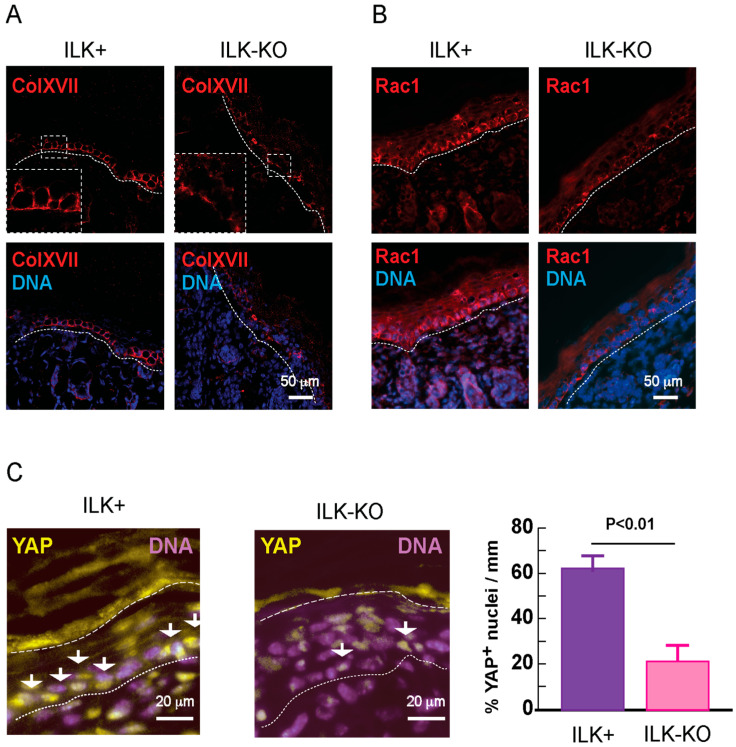
Abnormalities in Collagen XVII (ColXVII), Rac1 and YAP1 immunoreactivity in ILK-KO epidermis. Representative micrographs of skin sections from 3-day-old ILK+ or ILK-KO mice immunostained with antibodies against the indicated proteins. Nuclear DNA was visualized with Hoechst 33342. Dotted and dashed lines indicate, respectively, the dermal–epidermal junction and the outer edge of the epidermis. (**A**) Collagen XVII immunoreactivity. (**B**) Rac1 immunoreactivity. (**C**) YAP immunoreactivity, with arrows highlighting YAP-positive nuclei. The histogram in (**C**) represents the mean percentage of YAP-positive nuclei/mm skin + SD (Unpaired Student’s *t* test; *n* = 8 mice/genotype).

*Ilk* gene inactivation was also accompanied by alterations in Collagen XVII (BP180), a hemidesmosomal protein that binds to laminin 332 present at the dermoepidermal junction, and which contributes to epidermal attachment to the underlying basement membrane [16]. In a normal ILK+ epidermis, Collagen XVII immunoreactivity was detected in the basal and lateral aspects of basal keratinocytes. In contrast, in ILK-KO epidermis, we observed a discontinuous localization pattern of Collagen XVII immunoreactivity, with some areas lacking any detectable signal (Figure 2A). These alterations mirror disruptions in other cell–ECM adhesion components, including α6β4 and β1 integrins, which we have previously reported [7]. Thus, defective adhesion to the underlying basement membrane in ILK-deficient epidermis is associated with pronounced disruptions in focal adhesions and hemidesmosomal complexes.

ILK is necessary for proper Rac1 activation during directional migration in keratinocytes [5,7]. Rac1 modulates multiple epidermal functions, including maintenance of the stem cell compartments and wound healing [17,18]. In ILK+ epidermis, strong Rac1 immunoreactivity was readily detected throughout the entire cell body of all basal keratinocytes. In contrast, Rac1 immunoreactivity in some ILK-KO basal keratinocytes was not readily observed (Figure 2B).

Integrins and hemidesmosomal proteins, such as Collagen XVII, modulate many biological processes in keratinocytes, including proliferation, maintenance of stemness and responses to mechanical signals [19,20]. Given that those same biological processes are also modulated by the YAP transcriptional coactivator, we next compared its patterns of localization in epidermal tissues. In perinatal ILK+ epidermis, YAP immunoreactivity was observed in about 65% of basal keratinocytes and was restricted to the nuclei of these cells (Figure 2C). In contrast, nuclear YAP immunoreactivity was observed in only about 26% of basal keratinocytes in the ILK-KO epidermis (Figure 2C), suggesting the possibility that ILK functions as an upstream modulator of the YAP pathway in the epidermis.

### 2.2. Role of ILK in F-Actin Cytoskeletal Organization in Response to Mechanical Strain

**Figure 3:**
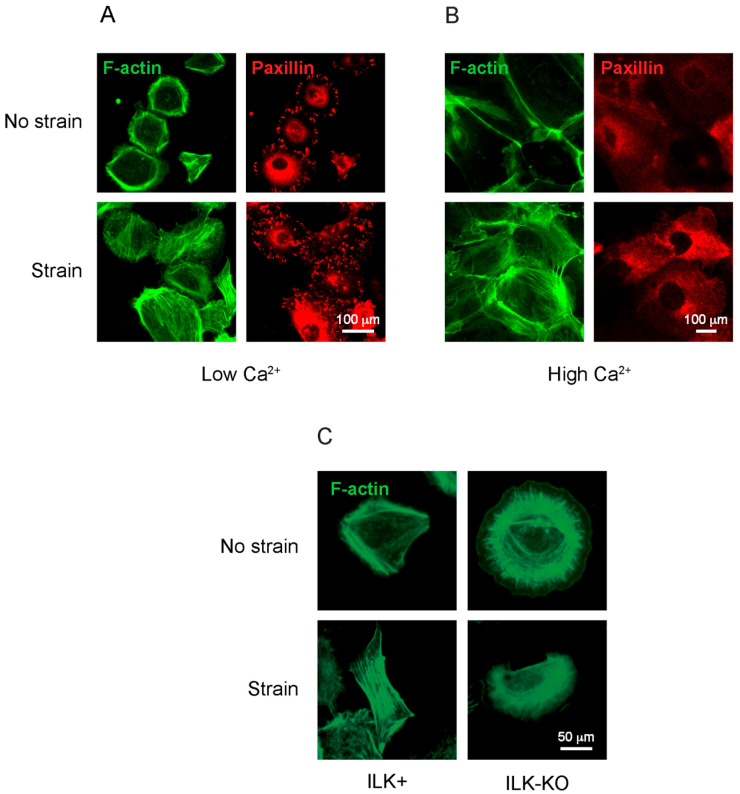
F-actin reorganization in response to mechanical strain. Representative photomicrographs of ILK+ keratinocytes cultured for 24 h in Low-Ca^2+^ medium (**A**) or High-Ca^2+^ medium (**B**), and then subjected to biaxial cyclical mechanical strain (20%; 0.1 Hz) for 16 h. The cells were processed for immunofluorescence microscopy to visualize paxillin immunoreactivity, or F-actin, using phalloidin. (**C**) ILK+ or ILK-KO keratinocytes were cultured in Low-Ca^2+^ medium and subjected to biaxial cyclical mechanical strain (20%; 0.1 Hz) for 16 h. Cells were processed for fluorescence microscopy, and F-actin was visualized using phalloidin.

The observed alterations in YAP nuclear immunoreactivity in ILK-deficient epidermis prompted us to next investigate the role of ILK in keratinocyte responses to mechanical stimuli in culture. Different types of mechanical load are present in various tissues in vivo, and keratinocytes are mainly exposed to stretch and to tensile strain [3]. Thus, we next cultured primary ILK+ keratinocytes in the presence of low extracellular Ca^2+^ concentrations (~50 μM, Low Ca^2+^) on collagen- and laminin 332-coated silicon membranes, which allow cell attachment and interactions with β1 integrins, and model basal keratinocytes in the epidermis. Under these conditions, the cells exhibited clearly visible focal adhesions at the cell membrane, as evidenced by paxillin immunoreactivity, as well as abundant cortical F-actin fibers and some stress fibers that ran through the entire cell body (Figure 3A). We also analyzed changes in F-actin organization and focal adhesions in cells subjected to a biaxial cyclic mechanical strain of 20%; 0.1 Hz, comparable to that present in some epithelia in vivo [21]. Following 16 h of strain, we observed noticeable increases in the number of focal adhesions per cell, and a reorganization of the F-actin cytoskeleton. Specifically, numerous thick actin bundles traversing the entire cell body were observed, with a concomitant apparent reduction in cortical actin filaments arranged parallel to the plasma membrane in many cells (Figure 3A).

We conducted similar experiments using cells that were cultured in the presence of high extracellular Ca^2+^ concentration (1.0 mM, High Ca^2+^) for 24 h, which model differentiated keratinocytes present in the suprabasal layers of the epidermis. In the absence of mechanical strain, F-actin undergoes reorganization in response to the elevation in extracellular Ca^2+^ levels, forming bundles that run parallel to the plasma membrane, and which are known to associate with adherens and tight junctions that contribute to the maintenance of sealed epidermal cell–cell borders [22,23]. Under these conditions, β1 integrin expression is downregulated [24] and, consequently, paxillin immunoreactivity is no longer observed at focal adhesions associated with the plasma membrane (Figure 3B). Significantly, numerous thick F-actin filaments were assembled throughout the cell body and along the cell periphery in those cultures that were additionally exposed to mechanical strain (Figure 3B). These observations are consistent with the notion that cyclical mechanical strain induces pronounced rearrangements in the F-actin cytoskeleton in cultured epidermal keratinocytes, irrespective of their differentiation status.

We next compared the effect of mechanical strain on F-actin reorganization between undifferentiated ILK-expressing and ILK-deficient keratinocytes. In the absence of mechanical strain, F-actin filaments in ILK-KO cells exhibit multiple abnormalities, including the formation of thick actin cables surrounding the nucleus, with finer perpendicular fibers (Figure 3C). This arrangement was largely maintained in those ILK-KO cells subjected to mechanical strain, and no cells exhibiting thick actin fibers running along the cell body were observed, in contrast to ILK+ keratinocytes (Figure 3C).

### 2.3. ILK Modulation of YAP Responses to Mechanical Strain

**Figure 4:**
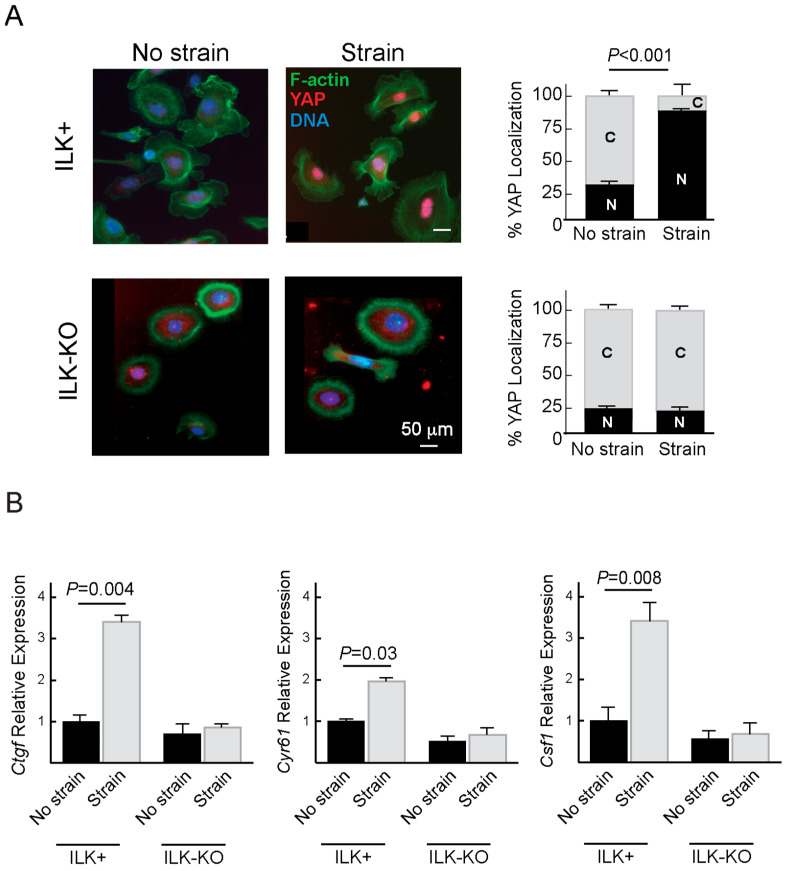
Mechanical strain induces YAP nuclear translocation and activation. (**A**) Keratinocytes of the indicated genotypes were cultured in Low-Ca^2+^ medium and then were subjected to biaxial cyclical mechanical strain (20%; 0.1 Hz) for 16 h. The cells were processed for immunofluorescence microscopy with anti-YAP antibodies. F-actin and DNA were visualized, respectively, using phalloidin and Hoechst 33342. The histograms on the right represent the percentage of cells of each genotype exhibiting YAP immunoreactivity concentrated in the nucleus (N) or in the cytoplasm (C). The data are shown as the mean + SEM, and the *p* values represent differences between nuclear as well as cytoplasmic fractions in cells subjected to strain, relative to non-strained cultures (Unpaired Student’s *t* test). (**B**) RT-qPCR analysis of *Ctgf*, *Cyr61* and *Csf1* mRNAs in keratinocytes cultured in the presence or absence of bidirectional cyclical strain (16 h, 20%, 0.1 Hz). The data are shown as the mean + SEM (2-way ANOVA; *n* = 5).

In epidermal and other cell types, YAP and TAZ function as mechanosensors downstream from the F-actin cytoskeleton [11]. The impairment in F-actin rearrangements in response to mechanical strain observed in ILK-KO keratinocytes prompted us to investigate whether YAP responses may also be affected in these cells. Analysis of the intracellular distribution of YAP immunoreactivity in subconfluent cultures of ILK+ cells cultured in Low-Ca^2+^ medium revealed a nuclear localization in about 25% of the cells, with the remainder exhibiting a predominantly cytoplasmic localization. Following 16 h of mechanical strain, the proportion of cells in which YAP immunoreactivity was nuclear increased to about 85% (Figure 4A). In contrast, the proportion of ILK-KO cells in which YAP was detected in the nucleus was about 17%, irrespective of whether or not mechanical strain was applied (Figure 4A).

We next investigated whether nuclear localization of YAP is associated with changes in the expression of its target genes. To this end, we determined the effect of mechanical strain on two well-established YAP targets, which have been associated with cellular responses to mechanical forces. Specifically, the expression of Ctgf and Cyr61 increases as a result of pathologic tissue stiffening [25]. We additionally examined the expression of Csf1, as it is upregulated in response to α3β1 integrin-mediated activation of YAP in epidermal keratinocytes, and ILK is a downstream modulator of responses to α3β1 integrins [26]. While we observed significant increases in the levels of Ctgf, Cyr61 and Csf1 transcripts in mechanically stimulated ILK+ keratinocytes, no significant changes in these transcripts were evident in ILK-KO cultures (Figure 4B). These observations are consistent with the notion that ILK is an important upstream modulator of YAP mechanoresponses in keratinocytes subjected to cyclical bidirectional strain.

### 2.4. ILK-Dependent Induction of MicroRNA Expression Changes in Response to Mechanical Strain

**Figure 5:**
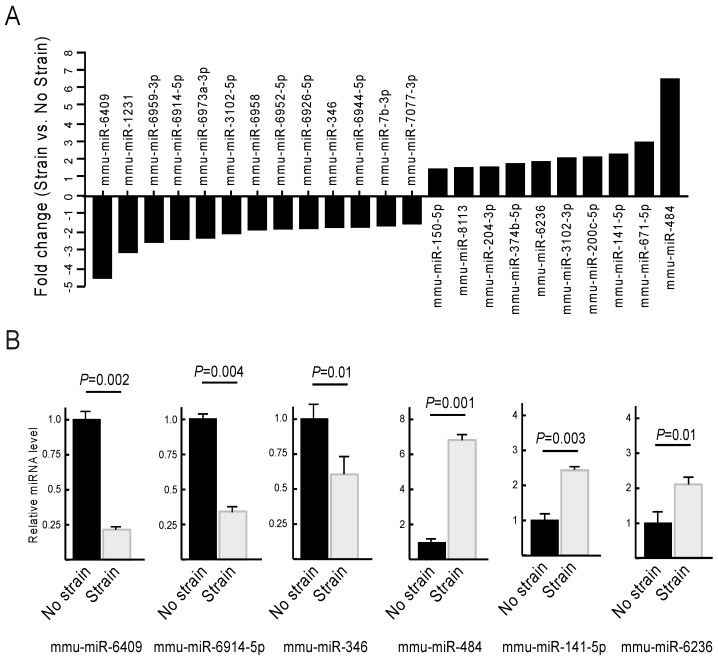
Identification of mechanosensitive miRNAs in ILK+ keratinocytes. (**A**) ILK+ keratinocytes were cultured in Low-Ca^2+^ medium and subjected to biaxial cyclical mechanical strain (20%; 0.1 Hz) for 16 h. mRNA was isolated and subjected to microarray analysis. The histograms show the mean magnitude of changes in the abundance miRNA species measured in cultures subjected to mechanical strain, relative to those in unstimulated cells (*n* = 3). (**B**) RT-qPCR analysis of the indicated miRNAs in ILK+ keratinocytes cultured in the presence or absence of bidirectional cyclical strain (16 h, 20%, 0.1 Hz). The data represent miRNA levels in cells subjected to mechanical strain relative to levels in unstimulated keratinocytes, set to 1.0. Data are shown as the mean + SEM (Unpaired Student’s *t* test; *n* = 4).

Cellular responses to mechanical stimuli are orchestrated through the activation of multiple pathways, including those modulated by microRNAs (miRNAs) [27,28]. For this reason, we next examined whether cyclical mechanical strain induced miRNA changes in keratinocytes. To this end, we conducted a microarray analysis of miRNA species present in ILK+ keratinocyte cultures in the presence and absence of mechanical strain. This analysis, coupled with miRNA annotations from the miRbase and miRGene databases, revealed 13 significantly differentially downregulated and 10 upregulated miRNAs in cells subjected to mechanical strain compared to unstrained cultures, with FC > 1.6 and an adjusted *p* value < 0.05 (Figure 5A and Supplemental Table S1). We further validated the differential expression of six selected miRNAs by RT-qPCR analysis (Figure 5B).

**Figure 6:**
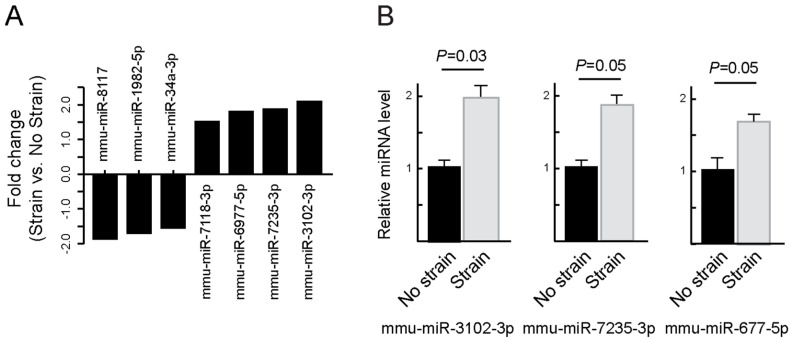
Identification of mechanosensitive miRNAs in ILK-KO keratinocytes. ILK-KO keratinocytes were cultured in Low-Ca^2+^ medium and subjected to biaxial cyclical mechanical strain (20%; 0.1 Hz) for 16 h. (**A**) mRNA was isolated and subjected to microarray analysis. The histograms show the mean magnitude of changes in the abundance miRNA species measured in cultures subjected to mechanical strain, relative to those in unstimulated cells (*n* = 3). (**B**) RT-qPCR analysis of the indicated miRNAs in ILK-KO keratinocytes cultured in the presence or absence of bidirectional cyclical strain. The data represent miRNA levels in cells subjected to mechanical strain relative to levels in unstimulated keratinocytes, set to 1.0. Data are shown as the mean + SEM (Unpaired Student’s *t* test; *n* = 4).

Analogous microarray analyses of ILK-KO cells subjected to strain identified seven differentially expressed miRNAs (three downregulated and four upregulated; Figure 6 and Supplemental Table S2). Notably, among these differentially expressed miRNAs identified, mmu-miR-3102-3p was the only species that was upregulated upon mechanical strain in both ILK+ and ILK-KO keratinocytes. This analysis further revealed several miRNAs upregulated by mechanical strain in an ILK-dependent manner, including mmu-miRNA-141-5p, mmu-miRNA-6236 and mmu-miRNA-8113.

**Figure 7:**
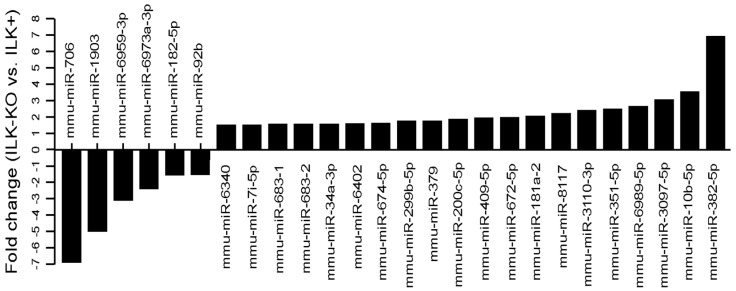
Identification of miRNAs differentially expressed in ILK-KO keratinocytes. ILK+ and ILK-KO keratinocytes were cultured in Low-Ca^2+^ medium in the absence of mechanical strain stimulation. mRNA was isolated and subjected to microarray analysis. The histograms show the mean magnitude of changes in the abundance miRNA species measured in ILK-KO cultures relative to those in ILK+ cells (*n* = 3).

We further assessed if there are differences in miRNA populations between ILK+ and ILK-KO keratinocytes not subjected to mechanical strain. This analysis identified six downregulated and 20 upregulated species in ILK-KO cells, compared to ILK+ cultures (Figure 7 and Supplemental Table S3). Together, these observations indicate that ILK is involved in modulating the levels of multiple miRNA species, both in unstimulated keratinocytes and in those cells subjected to cyclical mechanical strain.

## 3. Discussion

Integrins and their associated proteins, such as ILK, are key components of the mechanosensing and mechanotransduction machinery in metazoans. The present studies reinforce the concept that ILK is a fundamental regulator of epidermal biology, integrating mechanical cues sensed by integrins with biochemical signaling that controls keratinocyte behavior.

Our analysis of cutaneous tissues is consistent with the notion that epidermal *Ilk* inactivation results in a phenotype that shares characteristics with wound healing and inflammation, including activation of TGF-β pathways, likely arising from the loss of epidermal integrity. We and others previously reported the presence of blisters in perinatal mice with epidermis-specific *Ilk* gene inactivation, which were often observed at the dermal–epidermal junction in areas subjected to friction [5,7]. Notably, these animals also develop blisters during embryonic development, under conditions devoid of any substantial mechanical friction on the skin. This underscores the central role of epidermal ILK in the intrinsic maintenance of epidermal and cutaneous integrity.

At the cellular level, ILK is an essential component of integrin-containing focal adhesions. Focal adhesions in keratinocytes are mainly composed of integrins α3β1 and α2β1 [29]. In the intact epidermis, focal adhesions and hemidesmosomes crosstalk and modulate each other, contributing to epidermal adhesion to the underlying basement membrane and mechanosignaling [30,31]. In the absence of ILK, pronounced abnormalities occur in the distribution of hemidesmosomal α6β4 integrins at the dermal–epidermal junction [5,7]. We now report that hemidesmosomal defects in ILK-KO keratinocytes further extend to perturbations in Collagen XVII. Hemidesmosomes are critical for epidermal adhesion to the basement membrane, participating in the maintenance of cutaneous integrity and homeostasis [32]. Hemidesmosome assembly involves the initial association of β4 integrin cytoplasmic tails with plectin, followed by clustering. Once this complex is formed, Collagen XVII is incorporated through its ability to bind to both β4 integrin and plectin [33]. Significantly, inherited genetic alterations in focal adhesions or hemidesmosomes generate an altered keratinocyte microenvironment, characterized by the presence of inflammation and a wound-healing phenotype [34]. This phenotype is consistent with our observations in an ILK-deficient mouse epidermis, which shows upregulation of TGF-β-mediated responses, known to be essential for cutaneous wound repair. Our studies further establish ILK as a critical modulator of focal adhesion and hemidesmosome assembly in basal keratinocytes in vivo. ILK-deficient suprabasal keratinocytes additionally exhibit defects in adherens and tight junctions [8], thus placing ILK as a central contributor to the formation of multiple types of cell–ECM and cell–cell junctions in epidermal tissues.

ILK is an upstream regulator of Rac1 in various tissues. In the epidermis, ILK and Rac1 are involved in wound healing, and Rac1 also plays a role in the maintenance of keratinocyte stem cells [6,35,36]. The observed alterations in Rac1 immunoreactivity in basal ILK-KO cells suggest the possibility that dysregulation of Rac1 may also be involved in some of the alterations identified in the ILK-KO epidermis. Similarly, in cultured epidermal keratinocytes, Rac1 is activated in an ILK-dependent manner upon induction of forward migration and during KGF-mediated phagocytosis [7,37]. Significantly, Rac1 also contributes to responses to mechanical stimuli, and is activated in epithelial HeLa cells subjected to biaxial cyclical strain and during the development of mechanical tension associated with spreading over large surfaces [38]. Whether Rac1 is activated in an ILK-dependent manner in keratinocytes subjected to cyclical mechanical strain will be an important area for future research.

In vivo, epidermal maintenance and repair rely on keratinocyte stem cells and their transit-amplifying progeny, both located on the basal layer. Similarly, mechanical stretching of the skin promotes tissue expansion via activation of multiple pathways, including YAP-dependent mechanisms, which lead to increased keratinocyte stem cell renewal [39]. Keratinocyte α3β1 integrins mediate adhesion to laminin 332 in the underlying basement membrane [13], maintaining keratinocyte stemness. The latter is accomplished through mechanisms that involve nuclear translocation and activation of YAP, possibly through Hippo-independent mechanisms [40]. Integrin α3β1 modulates YAP activity in mouse keratinocytes through Src- and focal adhesion kinase (FAK)-related pathways [26]. ILK also mediates cellular responses to α3β1 stimulation, and the marked reduction we observed in the fraction of basal keratinocytes that display nuclear YAP localization in ILK-KO epidermis is consistent with the concept that ILK also likely participates in the maintenance of the keratinocyte stem cell population. In agreement with this proposal, ILK-KO keratinocytes also exhibit markedly decreased proliferative and regenerative capacity in vivo [6,7].

Mechanotransduction in the epidermis involves a complex interplay of integrins, focal adhesion proteins, RhoA, F-actin remodeling, and transcriptional regulators such as YAP/TAZ [41,42]. Integrins act as primary mechanosensors, transmitting extracellular matrix stiffness and strain signals to intracellular adhesion complexes. Downstream, FAK phosphorylation and RhoA activation drive actomyosin contractility and focal adhesion maturation, forming a feed-forward loop that reinforces mechanosignaling [2]. In cultured human keratinocytes, high tension conditions together with an intact F-actin cytoskeleton are necessary for YAP nuclear translocation, the inhibition of Notch signaling and the maintenance of the undifferentiated state [43].

A key finding of our work is the identification of ILK as a critical component of the integrin–F-actin–YAP regulatory mechanotransduction axis in response to cyclical strain in keratinocytes. In the absence of ILK, YAP fails to activate mechanosensitive target genes, indicating that ILK functions upstream of YAP in the mechanosignaling cascade. Collectively, these results position ILK as a critical node linking integrin adhesion complexes to cytoskeletal remodeling and transcriptional responses. In particular, the impaired transcriptional activation in response to mechanical strain of the YAP target genes *Ctgf*, *Cyr61* and *Csf1* in the absence of ILK may have implications for cutaneous regeneration after injury, as these mechanoresponsive factors play key roles in proper wound healing. This would also be consistent with the significant delays in cutaneous repair observed in the ILK-KO epidermis [6]. Given that ILK can also modulate RhoA activation [8], an important area for future research will be to determine the relationship between ILK, FAK/Src, RhoA and Notch in the biomechanical regulation of keratinocyte stemness.

Importantly, our data also reveal that ILK also influences the miRNA regulatory landscape. In unstimulated keratinocytes, ILK deficiency alters basal miRNA expression profiles, suggesting a role for ILK in maintaining homeostatic miRNA regulation. Further, mechanical stimulation by cyclical strain induces significant changes in miRNA expression in wild-type, but not in ILK-KO keratinocytes. This indicates that ILK is necessary not only for cytoskeletal and transcriptional responses, but also for the mechanosensitive miRNA network, which may fine-tune gene expression under mechanical stress.

MicroRNAs have emerged as important post-transcriptional regulators, and a given miRNA can target multiple genes involved in the same pathway or biological process. The ILK-dependent mechanosensitive miRNAs we identified include mmu-miRNA-141-5p, mmu-miRNA-6236 and mmu-miRNA-8113. The mmu-miRNA-141-5p species positively regulates inflammatory responses and cell migration, and it is a post-transcriptional regulator of *Tgfb2* [44]. mmu-miRNA-6236 plays modulatory roles in angiogenic responses associated with vascular tissue repair. Significantly, targets for mmu-miRNA-6236 include the platelet-derived growth factor alpha peptide (*PDGFA*), which is involved in endothelial cell proliferation and migration, relevant to wound healing, and p21 protein [Cdc42/Rac] activated kinase 2 (PAK2), which participates in F-actin dynamics [45]. Although little is known about the function of mmu-miRNA-8113, analysis of its putative targets suggests potential involvement in cell proliferation, apoptosis and responses to oxidative stress [46].

Given that several miRNAs modulate YAP activity and cytoskeletal regulators such as RhoA [47,48], these findings suggest a multilayered ILK-dependent mechanotransduction system integrating structural, transcriptional, and post-transcriptional mechanisms. Our findings also suggest potential crosstalk between ILK and G-protein–coupled receptor pathways, such as those activated by the Calcium Sensing Receptor CaSR. Although CaSR has not been conclusively shown to respond to mechanical stimuli, ILK is necessary for CaSR signaling to RhoA, which in turn modulates actin organization [8]. This raises the possibility that ILK integrates signals from both mechanical and biochemical stimuli to fine-tune keratinocyte responses. Dysregulated mechanotransduction contributes to pathological conditions, such as tumor formation and chronic inflammation, but the molecular links remain incompletely understood. Future important studies should dissect how ILK-dependent signaling interfaces with YAP activity and miRNA networks under physiological and pathological mechanical stress.

In conclusion, our work positions ILK as a pivotal node that links mechanical inputs to cytoskeletal remodeling, YAP-dependent transcription and mechanosensitive miRNA expression in keratinocytes. By demonstrating that ILK is required for F-actin fiber formation and YAP nuclear translocation under cyclical strain, and that ILK loss prevents mechanosensitive target gene activation, while reprogramming basal and strain-induced miRNA changes, we reveal a multilayered modulatory pathway that integrates structural, transcriptional, and post-transcriptional control of keratinocyte behavior. These insights may have relevance for wound healing and regenerative medicine, for the generation of tissue-engineered skin equivalents, which might be optimized by tuning ECM stiffness, ligand presentation, and cyclic strain to sustain keratinocyte progenitor function and expansion. Our findings might also be translatable in oncology and inflammatory dermatology approaches to temper aberrant ILK–YAP signaling or correct dysregulated mechano-miRNA networks that contribute to proliferation, invasion or chronic inflammation.

## 4. Materials and Methods

### 4.1. Mouse Strains

The generation and genotyping of mice with epidermis-restricted inactivation of the *Ilk* gene (*K14Cre;Ilk^f/f^* mice) have been described [7,49]. All animal experiments were approved by the University of Western Ontario Animal Care Committee (Protocols No. 2015-021 and 2018-169), in accordance with regulations and guidelines from the Canadian Council on Animal Care. Protocols No. 2015-021 and 2018-169 (which included the research question, key design features and analysis plan) were prepared and approved before the study began.

All studies were conducted on cutaneous tissues isolated from 3 to 4 different littermates of each genotype, and repeated with at least four different, independent litters. All animals were genotyped prior to tissue harvesting, and no animals were excluded.

### 4.2. Antibodies

Antibodies, their sources and application details are listed in Table 1.

### 4.3. Keratinocyte Isolation and Culture

Keratinocytes were isolated from 3-day-old *K14Cre;Ilk^f/+^* mice (ILK+ and ILK-expressing, with one functional *Ilk* allele) or *K14Cre;Ilk^f/f^* littermates (ILK-KO and ILK-deficient) and cultured as described in [37,50,51]. All experiments were conducted with cells cultured for 48–72 h after isolation, at 70–80% confluence.

### 4.4. Mechanical Stimulation of Keratinocytes

Biaxial multidirectional cyclic strain was delivered to keratinocyte monolayers using a MechanoCulture B1 device (MBC1; CellScale Biomaterials Testing, Waterloo, ON, Canada) on which a silicone membrane was mounted, as described in [52,53]. Specifically, keratinocytes were seeded onto two clear silicone membranes (0.005″ thickness, McMaster-Carr, Aurora, ON, Canada, 87315K71) that had been sequentially precoated with rat-tail Collagen type I (5 μg/cm^2^, 22 °C, 2 h; Corning, Bedford, MA, USA, 354236) and laminin 332 matrix harvested from G804 rat bladder carcinoma cell-conditioned medium (37 °C, 2 h) [51], to assist with cell adhesion. Forty-eight hours after seeding, an equibiaxial cyclic tensile strain was applied to one of the silicon membranes (20% change in radius) with a frequency of 0.1 Hz for 24 h. Cells from the same epidermal isolate cultured on the second silicon membrane were not subjected to mechanical strain, and served as a static, isometric, unstimulated control.

### 4.5. Immunohistochemistry and Fluorescence Microscopy

To detect α-smooth muscle actin, 7 μm paraffin-embedded epidermis sections were fixed in Zn buffer (0.1 M Tris-HCl, pH 7.8, 0.05% Ca(OAc)_2_, 0.5% Zn(OAc)_2_, and 0.5% ZnCl_2_), and subjected to high-temperature antigen retrieval, with 10 mM sodium citrate (pH 6.0), followed by incubation with anti-α-SMA antibodies (Millipore Sigma, St. Louis, MO, USA, A2547) using Vectastain Elite ABC kits (PK-6100) and ImmPRESS reagent (Vector Laboratories, Burlingame, CA, USA, MP-2400). Immunoreactivity signals were visualized with ImmPACT NovaRED peroxidase substrate (Vector Laboratories, Newark, CA, USA, SK-4805), and tissues were counterstained with Methyl Green. To detect all other proteins, tissues were protected by embedding in Optimal Cutting Temperature (OCT) compound (Sakura Finetek, Torrance, CA, USA, 4583) prior to freezing. Cryosections (7–10 μm) were fixed with 4% paraformaldehyde dissolved in phosphate-buffered saline (PBS). Fixed tissues were treated for 15 min with 0.2% Triton X-100 in PBS, followed by a 30 min blocking incubation with Powerblock Universal Blocking Reagent (BioGenex, San Ramon, CA, USA, HK085-5K). Following incubation with primary antibodies, samples were treated with appropriate Alexa Fluor^®^-labeled secondary antibodies (Table 1). Mouse-on-Mouse (M.O.M.) basic kits (Vector Laboratories, BMK-2202) were used with mouse monoclonal primary antibodies, following the manufacturer’s instructions. Each experiment that examined epidermal sections was conducted on tissues from 3 littermates of each genotype (ILK+ and ILK-KO). For each epidermis harvested from individual mice, 10 non-serial tissue sections were examined (3–4 microscopy fields per section).

Cultured keratinocyte specimens were processed for immunofluorescence microscopy, as described in [54]. For experiments using cultured keratinocytes, cells were isolated from 8 to 12 mice of each genotype (2–3 litters), and each experiment was conducted with 3–6 independent cell isolates. Fluorescence photomicrographs were obtained with a Leica DMIRBE fluorescence microscope (Leica, Wetzlar, Germany) equipped with an Orca-ER digital camera (Hamamatsu Photonics, Shizuoka, Japan), using Volocity 6.1.1 software (Improvision, Coventry, UK).

### 4.6. Immunoblot Analysis

To prepare epidermal lysates, dorsal skins from 3-day-old mice were harvested and incubated with Dispase II (Roche 4942078001; 8 mg/mL, final, and purchased from Millipore Sigma, St. Louis, MO, USA) for 1 h at 37 °C. The epidermis was mechanically separated from the dermis and homogenized in buffer A (100 mM Tris-HCl, pH 6.8, 1% NP-40, 10 mM EDTA, and 1 M urea). Proteins in cell lysates were resolved by denaturing polyacrylamide gel electrophoresis and analyzed by immunoblot with the antibodies listed in Table 1, as described in [54].

### 4.7. Microarray Analysis and Quantitative Reverse Transcription-Polymerase Chain Reaction (RT-qPCR)

RNA was isolated from primary keratinocytes cultured for 24 h in the presence or absence of mechanical stimulation, using miRNeasy Tissue/Cell Advanced Mini Kits (Qiagen, Markham, ON, Canada, 217604), following the manufacturer’s instructions. The concentration and purity of RNA were determined with an Agilent 2100 Bioanalyzer (Agilent, Santa Clara, CA, USA). Only samples with integrity numbers ≥ 9.0 were used. miRNA microarray analyses were conducted by the London Regional Genomics Centre, using Affymetrix miRNA 4.0 Arrays (Thermo Fisher, Waltham, MA, USA, 902412).

The expression levels of mRNAs were measured by RT-qPCR, as described previously [55]. qPCR was conducted on a CFX384 Real-Time PCR system (Bio-Rad, Hercules, CA, USA) operated by CFX Manager software (version 1.6). cDNA samples corresponding to 20 ng of RNA were amplified using PerfeCTa qPCR SuperMix (Quanta Biosciences, Gaithersburg, MD, USA, 95050-500). The following primers were used (Thermo Fisher Scientific, Waltham, MA, USA; 400 nM, final): *Ctgf*—forward 5′-GTGGAATATTGCCGGTGCA-3′, reverse 5′-CCATTGAAGCATCTTGGTTCG-3′; *Cyr61*—forward 5′-ATTCTTGAGTAGCATTAGG-3′, reverse 5′-GTACTATGAAGCGAAGTC-3′; and *Csf1*—forward 5′-GCCTCCTGTTCTACAAGTGGAAG-3′, reverse 5′-ACTGGCAGTTCCACCTGTCTGT-3′. The results were normalized to the expression of the housekeeping genes *Rpl16* and *Rps29*, which encode ribosomal protein L16 and S29, respectively [56]. Primer specificity and amplicon sizes were confirmed, respectively, by analysis of melt curves conducted between 65 °C and 95 °C, in 0.5 °C increments, and agarose gel electrophoresis. Relative mRNA levels were calculated using the ΔΔCt method.

The expression levels of miRNAs were determined using the miRCURY LNA miRNA SYBR Green PCR System (Qiagen, Toronto, ON, Canada, 339345), following the manufacturer’s protocol. miRNA expression levels were normalized to miR-103a-3p and calculated using the ΔΔCt method. Melting curves were generated after each RT-qPCR experiment to verify the quality of the amplification products. Primers for miRNA amplification were purchased from Qiagen (Toronto, ON, Canada). For all mRNA and miRNA qPCR experiments, replicate samples of at least three RNA preparations obtained from three different cell isolates were analyzed.

### 4.8. Statistical Analysis

All statistical analyses were conducted using GraphPad Prism (version 10.4.2, GraphPad Software Inc., La Jolla, CA, USA). Data were analyzed using Unpaired Student’s *t* test or two-way ANOVA, with post hoc Tukey’s correction, as indicated in individual experiments. Significance was set at *p* < 0.05. Experiments with cultured cells were conducted in triplicate samples, and with 3–5 independent cell isolates.

## Figures and Tables

**Table 1:** Antibodies used in this study.

Antibody	Dilution	Source
α6 integrin	IF 1:100 ^1^	
β1 integrin	IF 1:100	EMD Millipore, St. Louis, MO, USA (MAB1997)
α-Smooth muscle actin (SMA)	IF 1:500	EMD Millipore, St. Louis, MO, USA (A2547)
Phospho-SMAD 2	IB 1:1000	EMD Millipore, St. Louis, MO, USA (AB3849)
SMAD 2/3	IB 1:1000	BD Transduction Laboratories, Mississauga, ON, Canada (610842)
Paxillin	IF 1:100	BD Transduction Laboratories, Mississauga, ON, Canada (610051)
YAP	IF 1:100	Cell Signaling Technology, Danvers, MA, USA (14074S)
Glyceraldehyde 3-phosphodehydrygenase (GAPDH)	IB: 1:5000	Enzo Life Sciences, Farmingdale, NY, USA (ADI-CSA-335-E)
Collagen XVII		Abcam, Cambridge, UK (ab184996)
HRP-conjugated anti-mouse IgG	IB 1:5000	Jackson ImmunoResearch, West Grove, PA, USA (115-135-003)
HRP-conjugated anti-rabbit IgG	IB 1:5000	Jackson ImmunoResearch, West Grtove, PA, USA (111-035-144)
AlexaFluor^TM^ 488-conjugated anti-mouse IgG	IF 1:250	Thermo Fisher Scientific, Waltham, MA, USA (A-11001)
AlexaFluor^TM^ 594-conjugated anti-mouse IgG	IF 1:250	Jackson ImmunoResearch, West Grove, PA, USA (115-585-062)
AlexaFluor^TM^ 555-conjugated anti-rabbit IgG	IF 1:250	Thermo Fisher Scientific, Waltham, MA, USA (A-21428)

^1^ IB, immunoblot; IF, immunofluorescence microscopy.

## Data Availability

The original contributions presented in this study are included in the article/Supplementary Material. Further inquiries can be directed to the corresponding author.

## Supplementary Materials

The following supporting information can be downloaded at: https://www.mdpi.com/article/10.3390/ijms27062858/s1.

## Abbreviations

The following abbreviations are used in this manuscript:

ECM	Extracellular matrix
FAK	Focal adhesion kinase
ILK	Integrin-linked kinase
miRNA	microRNA
RT-qPCR	Reverse transcription-quantitative polymerase chain reaction
SEM	Standard error of the mean
TAZ	Transcriptional coactivator with PDZ-binding motif
TGF-β	Transforming growth Factor-beta
YAP	Yes-associated protein

## References

[1] Guo Y., Song Y., Xiong S., Wang T., Liu W., Yu Z., Ma X. (2022). Mechanical Stretch Induced Skin Regeneration: Molecular and Cellular Mechanism in Skin Soft Tissue Expansion. Int. J. Mol. Sci..

[2] Shutova M.S., Boehncke W.H. (2022). Mechanotransduction in Skin Inflammation. Cells.

[3] Reichelt J. (2007). Mechanotransduction of keratinocytes in culture and in the epidermis. Eur. J. Cell Biol..

[4] Górska A., Mazur A.J. (2022). Integrin-linked kinase (ILK): The known vs. the unknown and perspectives. Cell. Mol. Life Sci..

[5] Lorenz K., Grashoff C., Torka R., Sakai T., Langbein L., Bloch W., Aumalley M., Fassler R. (2007). Integrin-linked kinase is required for epidermal and hair follicle morphogenesis. J. Cell Biol..

[6] Nakrieko K.A., Rudkouskaya A., Irvine T.S., D’Souza S.J., Dagnino L. (2011). Targeted inactivation of integrin-linked kinase in hair follicle stem cells reveals an important modulatory role in skin repair after injury. Mol. Biol. Cell.

[7] Nakrieko K.A., Welch I., Dupuis H., Bryce D., Pajak A., St Arnaud R., Dedhar S., D’Souza S.J., Dagnino L. (2008). Impaired hair follicle morphogenesis and polarized keratinocyte movement upon conditional inactivation of integrin-linked kinase in the epidermis. Mol. Biol. Cell.

[8] Sayedyahossein S., Rudkouskaya A., Leclerc V., Dagnino L. (2016). Integrin-Linked Kinase Is Indispensable for Keratinocyte Differentiation and Epidermal Barrier Function. J. Investig. Dermatol..

[9] Morgner J., Ghatak S., Jakobi T., Dieterich C., Aumailley M., Wickstrom S.A. (2015). Integrin-linked kinase regulates the niche of quiescent epidermal stem cells. Nat. Commun..

[10] Judah D., Rudkouskaya A., Wilson R., Carter D.E., Dagnino L. (2012). Multiple roles of integrin-linked kinase in epidermal development, maturation and pigmentation revealed by molecular profiling. PLoS ONE.

[11] Elbediwy A., Vincent-Mistiaen Z.I., Spencer-Dene B., Stone R.K., Boeing S., Wculek S.K., Cordero J., Tan E.H., Ridgway R., Brunton V.G. (2016). Integrin signalling regulates YAP and TAZ to control skin homeostasis. Development.

[12] Totaro A., Panciera T., Piccolo S. (2018). YAP/TAZ upstream signals and downstream responses. Nat. Cell Biol..

[13] Pankratova M.D., Riabinin A.A., Butova E.A., Selivanovskiy A.V., Morgun E.I., Ulianov S.V., Vorotelyak E.A., Kalabusheva E.P. (2024). YAP/TAZ Signalling Controls Epidermal Keratinocyte Fate. Int. J. Mol. Sci..

[14] Abolhasani S., Ahmadi Y. (2025). MicroRNAs as Mediators of Mechanotransduction: From Fundamental Mechanisms to Therapeutic Applications. Cell Biochem. Biophys..

[15] Qin J., Wu C. (2012). ILK: A pseudokinase in the center stage of cell-matrix adhesion and signaling. Curr. Opin. Cell Biol..

[16] Van den Bergh F., Eliason S.L., Giudice G.J. (2011). Type XVII collagen (BP180) can function as a cell-matrix adhesion molecule via binding to laminin 332. Matrix Biol. J. Int. Soc. Matrix Biol..

[17] Benitah S.A., Frye M., Glogauer M., Watt F.M. (2005). Stem cell depletion through epidermal deletion of Rac1. Science.

[18] Tscharntke M., Pofahl R., Chrostek-Grashoff A., Smyth N., Niessen C., Niemann C., Hartwig B., Herzog V., Klein H.W., Krieg T. (2007). Impaired epidermal wound healing in vivo upon inhibition or deletion of Rac1. J. Cell Sci..

[19] Margadant C., Charafeddine R.A., Sonnenberg A. (2010). Unique and redundant functions of integrins in the epidermis. FASEB J..

[20] Turcan I., Jonkman M.F. (2015). Blistering disease: Insight from the hemidesmosome and other components of the dermal-epidermal junction. Cell Tissue Res..

[21] Le H.Q., Ghatak S., Yeung C.Y., Tellkamp F., Gunschmann C., Dieterich C., Yeroslaviz A., Habermann B., Pombo A., Niessen C.M. (2016). Mechanical regulation of transcription controls Polycomb-mediated gene silencing during lineage commitment. Nat. Cell Biol..

[22] Zarkoob H., Bodduluri S., Ponnaluri S.V., Selby J.C., Sander E.A. (2015). Substrate Stiffness Affects Human Keratinocyte Colony Formation. Cell. Mol. Bioeng..

[23] Vasioukhin V., Bauer C., Yin M., Fuchs E. (2000). Directed actin polymerization is the driving force for epithelial cell-cell adhesion. Cell.

[24] Jackson B.C., Ivanova I.A., Dagnino L. (2015). An ELMO2-RhoG-ILK network modulates microtubule dynamics. Mol. Biol. Cell.

[25] Rinschen M.M., Grahammer F., Hoppe A.K., Kohli P., Hagmann H., Kretz O., Bertsch S., Höhne M., Göbel H., Bartram M.P. (2017). YAP-mediated mechanotransduction determines the podocyte’s response to damage. Sci. Signal..

[26] Longmate W.M., Norton E., Duarte G.A., Wu L., DiPersio M.R., Lamar J.M., DiPersio C.M. (2024). Keratinocyte integrin α3β1 induces expression of the macrophage stimulating factor, CSF-1, through a YAP/TEAD-dependent mechanism. Matrix Biol. J. Int. Soc. Matrix Biol..

[27] D’Angelo A., Godeau A.L., Solon J. (2019). A New Player in Tissue Mechanics: MicroRNA Control of Mechanical Homeostasis. Dev. Cell.

[28] Lee D., Suh J., Jang Y., Suk M., Kim T.J. (2025). miR-548t-3p impairs nuclear mechanosensitivity and focal adhesion via lamin A/C downregulation. Biophys. J..

[29] Boulter E., Van Obberghen-Schilling E. (2006). Integrin-linked kinase and its partners: A modular platform regulating cell-matrix adhesion dynamics and cytoskeletal organization. Eur. J. Cell Biol..

[30] Pora A., Yoon S., Windoffer R., Leube R.E. (2019). Hemidesmosomes and Focal Adhesions Treadmill as Separate but Linked Entities during Keratinocyte Migration. J. Investig. Dermatol..

[31] Wang W., Zuidema A., Te Molder L., Nahidiazar L., Hoekman L., Schmidt T., Coppola S., Sonnenberg A. (2020). Hemidesmosomes modulate force generation via focal adhesions. J. Cell Biol..

[32] Zuidema A., Wang W., Sonnenberg A. (2020). Crosstalk between Cell Adhesion Complexes in Regulation of Mechanotransduction. Bioessays.

[33] Koster J., Geerts D., Favre B., Borradori L., Sonnenberg A. (2003). Analysis of the interactions between BP180, BP230, plectin and the integrin alpha6beta4 important for hemidesmosome assembly. J. Cell Sci..

[34] Wang Y., Hess M.E., Tan Y., Esser P.R., Nyström A., Boerries M., Sayar S.B., Has C. (2025). Alterations in the microenvironment of junctional epidermolysis bullosa keratinocytes: A gene expression study. Matrix Biol..

[35] DiPersio C.M. (2007). Double duty for Rac1 in epidermal wound healing. Sci. STKE Signal Transduct. Knowl. Environ..

[36] Takaya K., Imbe Y., Wang Q., Okabe K., Sakai S., Aramaki-Hattori N., Kishi K. (2024). Rac1 inhibition regenerates wounds in mouse fetuses via altered actin dynamics. Sci. Rep..

[37] Sayedyahossein S., Nini L., Irvine T.S., Dagnino L. (2012). Essential role of integrin-linked kinase in regulation of phagocytosis in keratinocytes. FASEB J..

[38] Phuyal S., Djaerff E., Le Roux A.L., Baker M.J., Fankhauser D., Mahdizadeh S.J., Reiterer V., Parizadeh A., Felder E., Kahlhofer J.C. (2022). Mechanical strain stimulates COPII-dependent secretory trafficking via Rac1. EMBO J..

[39] Aragona M., Sifrim A., Malfait M., Song Y., Van Herck J., Dekoninck S., Gargouri S., Lapouge G., Swedlund B., Dubois C. (2020). Mechanisms of stretch-mediated skin expansion at single-cell resolution. Nature.

[40] De Rosa L., Secone Seconetti A., De Santis G., Pellacani G., Hirsch T., Rothoeft T., Teig N., Pellegrini G., Bauer J.W., De Luca M. (2019). Laminin 332-Dependent YAP Dysregulation Depletes Epidermal Stem Cells in Junctional Epidermolysis Bullosa. Cell Rep..

[41] Wang J., Zhang Y., Zhang N., Wang C., Herrler T., Li Q. (2015). An updated review of mechanotransduction in skin disorders: Transcriptional regulators, ion channels, and microRNAs. Cell. Mol. Life Sci..

[42] Moro A., Driscoll T.P., Boraas L.C., Armero W., Kasper D.M., Baeyens N., Jouy C., Mallikarjun V., Swift J., Ahn S.J. (2019). MicroRNA-dependent regulation of biomechanical genes establishes tissue stiffness homeostasis. Nat. Cell Biol..

[43] Totaro A., Castellan M., Battilana G., Zanconato F., Azzolin L., Giulitti S., Cordenonsi M., Piccolo S. (2017). YAP/TAZ link cell mechanics to Notch signalling to control epidermal stem cell fate. Nat. Commun..

[44] Liep J., Kilic E., Meyer H.A., Busch J., Jung K., Rabien A. (2016). Cooperative Effect of miR-141-3p and miR-145-5p in the Regulation of Targets in Clear Cell Renal Cell Carcinoma. PLoS ONE.

[45] Mani A.M., Lamin V., Peach R.C., Friesen E.H., Wong T., Singh M.V., Dokun A.O. (2024). miRNA-6236 Regulation of Postischemic Skeletal Muscle Angiogenesis. J. Am. Heart Assoc..

[46] Tiwari N., Kumar V., Gedda M.R., Singh A.K., Singh V.K., Gannavaram S., Singh S.P., Singh R.K. (2017). Identification and Characterization of miRNAs in Response to Leishmania donovani Infection: Delineation of Their Roles in Macrophage Dysfunction. Front. Microbiol..

[47] Zhang Y., Wang Y., Ji H., Ding J., Wang K. (2022). The interplay between noncoding RNA and YAP/TAZ signaling in cancers: Molecular functions and mechanisms. J. Exp. Clin. Cancer Res..

[48] Liu M., Bi F., Zhou X., Zheng Y. (2012). Rho GTPase regulation by miRNAs and covalent modifications. Trends Cell Biol..

[49] Rudkouskaya A., Welch I., Dagnino L. (2014). ILK modulates epithelial polarity and matrix formation in hair follicles. Mol. Biol. Cell.

[50] Sayedyahossein S., Xu S.X., Rudkouskaya A., McGavin M.J., McCormick J.K., Dagnino L. (2015). Staphylococcus aureus keratinocyte invasion is mediated by integrin-linked kinase and Rac1. FASEB J..

[51] Dagnino L., Ho E., Chang W.Y. (2010). Expression and analysis of exogenous proteins in epidermal cells. Methods Mol. Biol..

[52] Demir T., Takada H., Furuya K., Sokabe M., Ogawa R. (2022). Role of Skin Stretch on Local Vascular Permeability in Murine and Cell Culture Models. Plast. Reconstr. Surg. Glob. Open.

[53] Kim M.K.M., Burns M.J., Serjeant M.E., Séguin C.A. (2020). The mechano-response of murine annulus fibrosus cells to cyclic tensile strain is frequency dependent. JOR Spine.

[54] Dutta A., Calder M., Lina D. (2025). Role of Kindlin-2 in cutaneous squamous carcinoma cell migration and proliferation: Implications for tumour progression. Int. J. Mol. Sci..

[55] Crawford M., Leclerc V., Dagnino L. (2017). A reporter mouse model for in vivo tracing and in vitro molecular studies of melanocytic lineage cells and their diseases. Biol. Open.

[56] de Jonge H.J., Fehrmann R.S., de Bont E.S., Hofstra R.M., Gerbens F., Kamps W.A., de Vries E.G., van der Zee A.G., te Meerman G.J., ter Elst A. (2007). Evidence based selection of housekeeping genes. PLoS ONE.

